# Supplementary material for: Combined Neutrophil-to-Lymphocyte and Platelet-Volume-to-Platelet Ratio (NLR and PVPR Score) Represents a Novel Prognostic Factor in Advanced Gastric Cancer Patients
Source: J Clin Med. 2021 Aug 30;10(17):3902. doi: 10.3390/jcm10173902 (PMC8432226; doi:10.3390/jcm10173902)
Supplement: Supplementary file 1 [file jcm-10-03902-s001.zip › Supl1.pdf]

Supplement 1 *Agreement between dichotomized inflammatory markers (Cohen's κ)*

	1	2	3
1. NLR	1		
2. PVPR	0.056	1	
3. PDW	0.156*	0.533**	1

* $p<0.05$ (bilateral), ** $p<0.01$ (bilateral) NLR- neutrophil to lymphocyte ratio, PVPR – medium platelets volume to platelets ratio, PDW – platelets distribution width
