# Supplementary material for: Combined Neutrophil-to-Lymphocyte and Platelet-Volume-to-Platelet Ratio (NLR and PVPR Score) Represents a Novel Prognostic Factor in Advanced Gastric Cancer Patients
Source: J Clin Med. 2021 Aug 30;10(17):3902. doi: 10.3390/jcm10173902 (PMC8432226; doi:10.3390/jcm10173902)
Supplement: Supplementary file 1 [file jcm-10-03902-s001.zip › Supl2.pdf]

Suplement 2 *Overall survival (OS) and progression free survival (PFS) according to NLR, PVPR and NLR+revPVPR*

			Median (months)	95% CI	p-value
OS	NLR	high	9.3	7.9-10.7	0.001
		low	13.6	11.4-15.8	
	PVPR	high	13.6	11.3-15.9	0.035
		low	9.3	7.7-12.0	
PFS	NLR	high	5.5	4.6-6.3	0.018
		low	7.3	6.4-8.2	
	PVPR	high	7.3	6.7-7.9	0.044
		low	5.5	4.1-6.8	
OS	NLR+rev PVPR	high	8.4	5.8-11.1	<0.001
		medium	10.5	8.8-12.1	
		low	15.9	13.5-18.3	

NLR – neutrophile to lymphocyte ratio, PVPR – medium platelets volume to platelets ratio
